# Aerospace medical association letter to the editor

**DOI:** 10.1186/s12940-017-0342-1

**Published:** 2017-12-18

**Authors:** Philip J. Scarpa, Jeffrey C. Sventek

**Affiliations:** 1 Aerospace Medical Association Pilot Mental Health Working Group Chairperson, Cocoa Beach, Florida, USA; 2 Aerospace Medical Association Executive Director, Alexandria, Virginia USA

Dear Editor,

Thank you for publishing the article “Airplane pilot mental health and suicidal thoughts: a cross-sectional descriptive study via anonymous web-based survey” by Wu, et al., [1] 15 December 2016.

We are glad the authors chose to investigate the area of pilot mental health and recognized the need to acquire data on the issue directly from sampling pilot populations. The literature on pilot mental health is insufficient and greater study is needed.

We also acknowledge and appreciate the authors pointing out in the article the limitations of methodology and sampling within this study and the results and conclusions should be viewed with this in mind. Unfortunately, the press release on the article omitted this important information.

The mental health and well-being of pilots is a very important area and one that the Aerospace Medical Association has been investigating and promoting for decades [2]. Our Association recommends that mental health should always be evaluated as part of the aeromedical assessment of pilots, that emphasis should be given to the less serious and more common mental health conditions that are more likely to be predictable and responsive to treatment, that greater rapport and trust be established between the aeromedical examiner and pilot, that “safe zones” for reporting by pilots be set up and utilized, and that training and greater awareness of the examiner and in the aviation community is key to the success and effectiveness of these recommendations. We also encourage guidelines be established, if none exist, on substance abuse/misuse (based on the ICAO Manual on Prevention of Problematic Use of Substances in the Aviation Workplace), [3] and on healthcare provider reporting versus patient confidentiality.

These recommendations were cited in the Germanwings Flight 9525 accident investigation report conducted by BEA, [4] and were published in our Association’s journal and on the Aerospace Medical Association website at https://www.asma.org/publications/aerospace-mental-health. We encourage all involved in aviation and in the health and well-being of pilots to read the full recommendations.

Thank you.

Working Group on Pilot Mental Health.

Aerospace Medical Association.

Philip J. Scarpa, MD, Chair.
