# Supplementary material for: Aerospace medical association letter to the editor
Source: Environ Health. 2017 Dec 18;16:132. doi: 10.1186/s12940-017-0342-1 (PMC5735864; doi:10.1186/s12940-017-0342-1)
Supplement: Related texts

# Reply: Response to Aerospace Medical Association Letter to the Editor

**Authors:** Alexander C. Wu, Deborah Donnelly-McLay, Marc G. Weisskopf, Eileen McNeely, Theresa S. Betancourt, Joseph G. Allen

**Affiliations:** 3 000000041936754X grid.38142.3c Department of Environmental Health, Harvard T.H. Chan School of Public Health, 665 Huntington Avenue, Building 1, Room 1301, Boston, MA 02115 USA; 4 000000041936754X grid.38142.3c Department of Global Health and Population, Harvard T.H. Chan School of Public Health, 665 Huntington Avenue, Building 1, Room 1104, Boston, MA 02115 USA; 5 000000041936754X grid.38142.3c Harvard T.H. Chan School of Public Health, 401 Park Drive, Landmark Center, 404-L, Boston, MA 02215 USA

We would like to thank the Working Group on Pilot Mental Health of the Aerospace Medical Association for their letter to the Editor and for their continued work on this important issue. We agree with their recommendations. Specifically, we feel there is an urgent need for further research and data collection on pilot mental health. We also agree with establishing “safe zones” for pilots to report mental health issues and receive help. We believe that this will reduce the barriers of stigma and fear of professional repercussions stemming from seeking help. We hope our study continues to increase awareness of depression and suicidal thoughts among airline pilots.
